# News and Miscellany

**Published:** 1881-07

**Authors:** 


### NEWS AND MISCELLANY.

Pleuro-Pneumonia has been stamped out of Pennsylvania.

A New Veterinary School.—It is proposed to start a new veterinary college at Aberdeen, Scotland.

Flaxseed Meal is recommended now for stock as superior to oil meal, because it can be gotten purer, and is quite as nutritious.

The Alumni of Columbia Veterinary College have organized into an association. It seems likely to become a very useful society.

A Cross-eyed Mule.—Dr. A. A. Holcombe, in the *Veterinary Review*, reports a case of a mule with double internal permanent strabismus. Quite a rare affliction.

Another Fertile Mule.—An army mule in Texas, not long ago, became pregnant from a mustang, and gave birth to a colt. The colt did not live, however.

New Veterinary Publication.—Mr. W. R. Jenkins is to republish in this city Gamgee's Veterinarian's Vade-Mecum, a work which has been quite popular in England.

Steel on Diseases of the Ox.—We have just received from John Wiley & Son, publishers, of this city, Steel's valuable work on the Ox. A full notice of it will be given in our next issue.

At the Veterinary Hospital at Leipsic, under charge of Dr. Zürn, inflammation of the legs is treated by putting the leg in a kind of long, thin rubber bag, which is kept filled with cold water.

Bi-chromate of Potassa.—Mr. Coultard praises this for femoro-tibial synovitis in cattle. It is recommended, first, to subdue active inflammation by cold; then apply the potassa, mixed with lard, in proportion of 4 to 30.

A New Cause for Pleuro-pneumonia.—Dr. Rose, V. S., of Richmond County, Staten Island, claims to have discovered in the lungs of a cow that died of pleuro-pneumonia a parasite, which he believes is the cause of the disease.

The Fluke Parasite is now found to have been widely spread in England by the inundations which were so prevalent and extensive in the late summer and autumn months of 1880, and flockmasters are once again in trouble.

An Exportation of Foot-and-Mouth Disease.—Nearly all the cattle by the Allan Line steamer "Phenician," which arrived at Glasgow May 31, from Boston, were found to be affected with foot-and-mouth disease. The slaughtered carcasses were boiled down.

Anthrax in the West.—Anthrax has been extensively prevailing among the cattle of the West, especially in Nebraska and Iowa. The Wyoming Cattle Association has commissioned Dr. Foote to investigate this disease, and also to inquire into pleuro-pneumonia.

The Horses of New York.—It is estimated that there are 50,000 horses in New York city. An exact census of them has never been taken. The average value of a horse is $200, and the stock, therefore, represents $10,000,000. The death rate among them is very high.

Equina, or Horse-pox, versus Phagedæna.—The disease which is described by Dr. Bowler as phagedæna is asserted by Dr. J. I. Tucker to be genuine "grease," or horse-pox. The facts given by Dr. Bowler seem to contradict this view, but the question cannot yet be decided.

A Horse in Spectacles.—Lord Denman, a friend of the domestic quadruped creation, drives a horse that wears spectacles about the streets of London. The animal was found to be near-sighted, and its owner has successfully tried the experiment of remedying the defect in the same way as is done with human beings.

Contagious Pleuro-Pneumonia in England still prevails, in spite of stringent measures to stamp it out. It is asserted that a great deal of the disease comes from infected cows in Ireland; and Ireland, instead of America, is now the country against which restrictive laws regarding importation are urged.

Veterinary Congresses.—A Veterinary Congress of the veterinarians of Belgium was held not long ago at Brussels, and was a very successful affair.

The British Veterinary Congress mentioned in our last issue will probably be held on July 13th and 14th, at London.

The Treatment for Dysentery in Dogs, at the Alfort Veterinary School, consists in giving subnitrate of bismuth and laudanum; and keeping the animal on two quarts of milk, sufficient water, and a drachm of bicarbonate of soda per day. This gives excellent results in the dysenteries that so often complicate sickness in young dogs.

Deaths.—The oldest teacher of veterinary medicine, Dr. E. Hering, late Director of the Stuttgard Veterinary School, died recently at the age of 82 years, from an attack of cerebral congestion, which carried him off in four days. He is best known by his Manual of Operative Veterinary Surgery.

Prof. Corbyn, one of the oldest veterinary practitioners of Philadelphia, died lately at the age of 72.

The Veterinary Surgeon's Bill, introduced last winter into the English Parliament, aims to compel all practicing veterinarians to become registered. It has been made a government measure, and will, undoubtedly, become a law. This will greatly raise the status of the English veterinarian. And he will probably be more disdainful of his American brother than ever.

But plumbers and druggists are registered in this country, "and why not every man?"

A Veterinary Law in Illinois has at last been enacted. It provides for the appointment of a State Veterinarian, who is empowered to keep watch upon and quarantine all cases of infectious disease among cattle. It compels persons also to notify the State Veterinarian of any case of pleuro-pneumonia among cattle upon his premises. The law is somewhat like that in New York State, but it seems to be a more complete one in some respects. Only $8,000, however, are appropriated for carrying it out.

American Pork.—A writer in the *Journal de Pharmacie et de Chimie* has found the following parasites in American pork, viz.: Tricocephalus dispar, Stephanurus dentatus, Echinorhyncus gigas, Cisticercus cellulosa, Fasciola hepatica, and Distomum lanceolatum. He also asserts that Trichina spiralis is not uncommon in American hams.

The gentleman who found all these things in American hams ought to settle in Chicago. He would get such a welcome!

Cribbing is a habit of vice which is acquired, usually in young animals when teething, or in horses of maturer age that are either too little used or too highly fed. It may arise from a morbid feeling induced by indigestion, or be learned by companionship. When the habit is acquired, there seems to be a fascination about it that defies the art of man to cure. We prevent it from day to day by neck-straps or muzzles, or patent halters or zinc hearts attached to five-ringed halters, or by pasting obnoxious materials or drugs upon the mangers, &c. But the desire, the habit, we fail to eradicate.

The Destruction of Lice.—Wm. Home, V.S., in the *Country Gentleman*, recommends the following as safe, and very effectual for the destruction of lice on cattle, horses and dogs.

Arsenious acid, two drachms.
Aqua, one pint.
M.

With a small sponge, the parts infested are to be well rubbed over with this mixture. The preparation to be constantly agitated, to prevent the arsenic settling to the bottom of the dish.

Treatment for Diarrhœa in Calves.—In the treatment of this, as of other forms of diarrhœa, the first step is to clear away the irritant from the bowels by a dose of 2 oz. castor oil and a teaspoonful of laudanum. It may be beneficial to add 5 drops of carbolic acid. When this has operated the patient may take, thrice a day 5 grains of the following mixture: Calomel 1 drachm, chalk 1½ oz. He may further take, three or four times daily, 2 teaspoonfuls tincture of ginger, 20 grains gum arabic, 2 drachms chalk, and 5 drops carbolic acid. A sixth of a rennet may be steeped in a quart of wine, and a tablespoonful given with every meal.

A Monstrosity in Fowls.—A full-grown chicken which was brought to the market of Shelbyville, Tenn., was found to possess three legs. A *post-mortem* examination, made by Dr. Fite, revealed the fact that the internal economy was even more queer than the extra leg. The crop, heart and lungs were natural, but the intestine, about midway of its length, subdivided into four distinct canals; these finally became reunited into one, and this, just before emerging from the body, divided into two distinct vents. The chicken was found also to have two distinct livers, one on each side. The monstrosity was a fat and healthy looking subject.

Museums for Private Veterinary Practitioners.—The establishment of small or large museums by private veterinary practitioners is a very useful suggestion that has recently been made by Dr. J. H. Steele, in the *Veterinary Journal.* Such a thing is very practicable, and should be attempted especially by the younger part of the profession. These museums should include anatomical specimens of all kinds, special devices for surgical treatment, models, dried preparations, charts, chemicals, etc. They might be made to illustrate any specialty which the veterinarian was engaged in. They would serve to educate pupils, illustrate cases, exhibit the methods of the practitioner, and would assist him in numerous ways.

Sows Eating Pigs.—A writer in the *Country Gentleman* gives the following as a preventive of the destructive habit:—When I have an old sow (they are the worst) ready to drop her pigs, I go to the smoke-house, get an old piece of refuse meat—an old piece with bone is the best, as they like to gnaw at a bone—I take this and give to the sow. Her appetite seems to crave something of the kind, and it is sometimes surprising to see how greedily she will devour the meat. I have seen sows leave corn, slops and pigs for the old salty meat. My father tried this several years ago, when other things had failed, and one piece of meat, say four or five pounds, seemed to satisfy her appetite, and she raised the pigs without any more disposition to eat them.

An Epidemic among Deer, resulting already in the death of more than one hundred valuable animals, has broken out among the deer herds in the Duke of Portland's park in Nottinghamshire, England. More deer are dying daily, and at present no means have been found to stop the spread of the infection. A *post-mortem* examination of one of the bodies has resulted in finding a small, thread-like worm infesting the bronchial tubes of the lungs and small intestines, the irritation of which caused congestion and inflammation of both organs, the symptoms being aggravated by the late prolonged wet and severe weather. The parasites are white, no thicker than a hair, and an inch long. It is reported that a similar disease has broken out in other English deer parks.

---

The Bill to Prevent the Spread of Contagious Diseases Among Animals passed the New York Legislature, but excited the following criticisms:

Mr. Jacobs opposed the passage of this bill, and read from the report of the Cattle Commissioners to show that the money appropriated for a similar purpose last year was squandered in many cases. He said healthy cows were killed and paid for at the rate of $15 per head, and their carcasses exposed for sale in market the next day. As a sample of extravagance, he cited one case where it cost the State $300 to kill one cow. Reckless expenditures were also shown in traveling expenses, hotel fare, carriage hire, &c., and he argued that no more money ought to be intrusted to the charge of the same officers who squandered last year's appropriation. The bill appropriates $50,000.

A Dog Home and Hospital.—There are several of these institutions in this city. An account of one of the largest, on Green street, was given in the *Tribune* not long ago. The "Home" contains about 300 dogs, only a small part being in the hospital wards. The keeper of the house and his wife have been in the business for thirty-five years. The wife is herself a dog doctress, and gets $3 for each of her visits. The proprietor says:

"People, somehow or other, will go to any sacrifice to save a pet dog, while they seldom scruple to kill a good horse that has broken its leg or become otherwise injured."

"Ay, it's a dire shame that so many fine horses should be slaughtered who only require a good surgeon to cure them completely. I have set horses' as well as dogs' limbs in such a manner that no expert would have been able to tell the difference."

According to reports, dog-doctoring in New York is a profitable business. It is a misfortune that it is so entirely in the hands of ignorant persons.

An Epidemic among the Horses at Chicago and Elsewhere.—A very malignant form of disease has prevailed among the horses of Chicago, and a large percentage, varying from 20 to 70 of the number, were at the time, in consequence, unfitted for work, and were kept in the barn for treatment. It first appears in the feet, just above the hoof, and develops very rapidly. A horse may go into the barn or stable at night with scarcely anything apparently ailing him, and be found the next morning unable to move from the stall. The first indication is a swelling, followed by a suppuration, which breaks out, producing a kind of running sore. As the disease grows the legs become swollen, and sometimes it spreads to the body, and when this occurs it is likely to result fatally. The cause is attributed to two sources. Some claim that the salt sprinkled on the car-track during the past winter is responsible for the disease. The other cause is said to be the long season of snow, ice, and frost, with frequent intervals of rain or moisture, in which the feet have stood while on the street or at work.

Later reports show that the disease has extended to Cleveland, O., Cincinnati, Milwaukee, Wis., and other places.

A full account of it is given by Dr. Bowler on page 199 of the Journal.

The Healthfulness of American Swine.—In view of the action taken by the French and other European governments in regard to American pork, as well as to be able to correct by positive and personal evidence the exaggerated reports which are published in Europe concerning hog cholera and trichinæ among American swine, Secretary Blaine sent the Chief of the Bureau of Statistics of the Department of State to Chicago and Cincinnati to investigate the entire question of hog-raising and pork-packing in the West in all its phases, "from the farm to the ship." In accordance with the Secretary's instructions, this gentleman visited representative hog-raisers, buyers, shippers, packing houses, stock yards, rendering establishments, health offices, and forwarding agents.

His report, which was submitted in May, was to the effect that American swine are the purest in breed and most healthy of any animals of the kind.

The report is received with some incredulity abroad, partly because it is, after all, only quasi-official, and represents but one man's investigations and opinion, and he a non-expert. Still, there is a much better feeling in Europe now regarding American pork.

Inversion of the Vagina of a Cow.—A case of this kind is related in the *Country Gentleman* as follows:

"My cow is three years old and is farrow. A few weeks ago I noticed something protruding from the vulva, looking hard, fleshy, and very red. This occurred when lying down. She seems to eat well, and gives a good quantity of milk. When standing, there is nothing to be seen, except there seems to be considerable inflammation outside."

The treatment advised by Dr. Moore is, to foment the protruded part with 4 qts. of warm water, and alum 6 oz., then return it with the hand easily. Put a single harness saddle on her and buckle one of the driving reins in the terret of the left side, then take the rein down that side under the belly and between udder and left hind leg, up the left side of tail and over back, tying in the right terret. Do the same with the other rein, which, of course, follows the right side and ends at the left terret. Thus the two cross over the spine. Then tie two strings connecting the reins, one near the top, the other near the bottom of the vulva. This will act as a truss. Give her the following drench: Sulphate of soda, 1¼ lb.; powdered saltpetre, 2 oz.; powdered ginger, 1 oz.; powdered capsicum, ½ dr., mix, dissolve in hot water and give at one dose. Inject, with a rubber syringe, three times daily, the following into the vagina: Tincture of opium, 1 oz.; elixir pro., 2 oz.; powdered alum, 2 oz.; water, 1 qt.; mix.

Elephant Dentistry at the Zoo.—One of the Indian elephants some time since had the misfortune to wrench off a portion of its trunk which had got caught in a noose of rope, and the largest African specimen, whose huge proportions are well known to the frequenters of the Gardens, met with an accident by which its tusks were broken off; the stumps subsequently grew into the cheeks, causing it excruciating pain, and necessitating an immediate remedy. The intrepid Superintendent undertook to perform the delicate operation and relieve the poor beast. Having prepared a gigantic hook-shaped lancet, he bandaged the creature's eyes and proceeded to his task. It was an anxious moment, for there was absolutely nothing to prevent the animal killing his medical attendants upon the spot, and to rely upon the common sense and good nature of a creature weighing many tons and suffering from facial abscesses and neuralgia, argues, to say the least of it, the possession of great nerve. But Mr. Bartlett did not hesitate, and climbing up within reach of his patient he lanced the swollen cheek. His courage was rewarded, for the beast at once perceived that the proceedings were for his good, and submitted quietly. The next morning, when they came to operate upon the other side, the elephant turned his cheek without being bidden, and endured the second incision without a groan.

A Case of Superfetation in the Horse.—A few cases of superfetation have been collected, but they are so rare as to make the following one very interesting. It was reported in the Lyons *Journal de Medicine Veterinaire.* A mare about ten years old gave birth, at full time, to a foal. Soon after the throes of parturition recommenced, and with assistance another foal was brought into the world. Twin foals are sufficiently rare to cause astonishment at any time, but the astonishment of the attendants was changed into amazement when they discovered that it was not a horse foal they had taken from the mare, but an unmistakable mule. The little creature, unfortunately, and as is generally the case with twins in solipeds, did not long survive its birth. The veterinary surgeon who was subsequently called in, made inquiry into the case, and as a result he gives the following explanation: It is a very common practice in Dauphiny to present the mare, when in œstrum, twice to the stallion, and at a brief interval, believing that fecundation is more certain by this procedure. But, as it seldom happens that the villagers can obtain the services of a stallion which can serve the mare twice with only a few minutes between, or yet a second stallion, and as male asses are by no means scarce, they oftentimes employ one of the latter to succeed the horse. And this is what happened with the mare alluded to. The case is not absolutely unique, even in equine physiology, however, for Fleming, in his "Text-book of Veterinary Obstetrics" (p. 152), gives six similar instances, and one in which a mare, put first to an English stallion, and soon after to a Barb, produced twin foals—one resembling the first, and the other the second stallion. It may be noted that, of all the domestic animals, the mare is the least disposed to multiparity.

